# Baseline HBsAg quantitative and CD4 T cell counts are associated with HBsAg loss in people living with HIV/HBV coinfection after combined antiretroviral therapy

**DOI:** 10.3389/fcimb.2025.1381826

**Published:** 2025-02-19

**Authors:** Muye Xia, Yuhang Zhao, Tao Yu, Xiaoli Lin, GuiChan Liao, Yuanhui Jiang, Jingchun Mao, Jie Peng, Shaohang Cai

**Affiliations:** ^1^ Department of Infectious Diseases, Nanfang Hospital, Southern Medical University, Guangzhou, China; ^2^ State Key Laboratory of Organ Failure Research, Guangzhou, China; ^3^ Key Laboratory of Infectious Diseases Research in South China (Southern Medical University), Ministry of Education, Guangzhou, China; ^4^ Guangdong Provincial Key Laboratory for Prevention and Control of Major Liver Diseases, Guangzhou, China; ^5^ Guangdong Provincial Clinical Research Center for Viral Hepatitis, Guangzhou, China; ^6^ Guangdong Institute of Hepatology, Guangzhou, China; ^7^ Guangdong Provincial Research Center for Liver Fibrosis Engineering and Technology, Guangzhou, China

**Keywords:** hepatitis B virus, HIV infection, CD4 T cell counts, HBsAg loss, HBsAg

## Abstract

**Background:**

Achieving Hepatitis B surface antigen (HBsAg) loss is a significant goal for chronic hepatitis B patients. This study aims to evaluate HBsAg loss in individuals with HIV/HBV coinfection and explore the association of clinical variables with this outcome.

**Methods:**

We enrolled 138 individuals coinfected with HIV/HBV and 480 HBV mono-infected individuals who initiated antiviral treatment. We employed Kaplan-Meier analysis to compare the rate of HBsAg loss between individuals with HIV/HBV coinfection and those with HBV mono-infection. In the HIV/HBV coinfected cohort, we used Cox proportional hazards models to assess the association between various factors and the incidence of HBsAg loss.

**Results:**

The cumulative HBsAg loss rate was higher among HBV/HIV coinfected individuals (13 patients, 11.5% at year 3) compared to HBV mono-infected individuals (1 patient, 0.6%) after antiviral therapy. In the HIV/HBV coinfected cohort, the multivariable analysis revealed that lower baseline HBsAg level (HR 0.53; 95% CI 0.38-0.74, p<0.001) and baseline CD4 T cell counts < 180 cells/uL (HR 0.32; 95% CI 0.10-0.96, p=0.042) were associated with an increased indicator of HBsAg loss. Additionally, the receiver-operating characteristic curve analysis indicated an area under the curve of 0.771 for baseline HBsAg levels and 0.758 for baseline CD4 cell counts at year 1 in predicting HBsAg loss.

**Conclusions:**

After antiretroviral therapy, HIV/HBV coinfected adults achieve higher rates of HBsAg loss. Baseline HBsAg quantitative and CD4 T cell counts are associated with HBsAg loss in individuals with HIV/HBV coinfection after combined antiretroviral therapy and may inform treatment decisions.

## 1 Introduction

Worldwide, between 5.1 to 12.4% of people living with HIV (PLWH) also have chronic hepatitis B virus (HBV) coinfection (Leumi et al., 2020). The most commonly recommended combined antiretroviral therapy (ART) for treating individuals with HIV/HBV coinfection is the combination of tenofovir disoproxil fumarate (TDF) or tenofovir alafenamide (TAF) with lamivudine (3TC) or emtricitabine (FTC). Due to its dual activity against HBV and HIV-1 infections, it has improved the control of HBV viremia and reduced liver fibrosis and drug resistance (Boyd et al., 2021; Ryom et al., 2022). In comparison to chronic HBV mono-infection, the presence of HIV in patients with HBV speeds up the advancement of chronic HBV to liver cirrhosis, hepatocellular carcinoma (HCC), or end-stage liver disease (Singh et al., 2017; Kouamé et al., 2018).

The loss of hepatitis B surface antigen (HBsAg), whether with or without developing antibodies to the surface antigen, is commonly considered a functional cure and the ultimate therapeutic goal for chronic hepatitis B (CHB) infection (European Association for the Study of the Liver, 2017; Martin et al., 2022). Nevertheless, this outcome is only attained in a small number of patients (Zhou et al., 2019; Hsu et al., 2021; Hsu et al., 2022). In recent times, a number of studies have indicated a greater occurrence of HBsAg seroclearance in individuals with HIV/HBV coinfection who undergo combined antiretroviral therapy (cART) in comparison to those with chronic HBV mono-infection (Yeo et al., 2019; Audsley et al., 2020; Chihota et al., 2020). Nevertheless, there are limited studies on the decline in HBsAg and the factors influencing it in HIV/HBV coinfection. It is essential to comprehend the potential predictors and biological markers linked to the loss of HBsAg in individuals suffering from HIV/HBV coinfection. This will enhance our understanding of the underlying mechanism of HIV/HBV coinfection and potentially aid physicians in formulating more efficacious treatment strategies. In this paper, we conducted a prospective examination of HBsAg loss following the initiation of cART in individuals with HIV/HBV coinfection. Additionally, we explored the association of clinical variables with this outcome.

## 2 Materials and methods

### 2.1 Study setting and participants

HIV/HBV Coinfected Cohort: Participants were selected from a longitudinal study (ChiCTR2200064212) on HIV/AIDS conducted at Nanfang Hospital (Southern Medical University, Guangzhou, China). The inclusion criteria for the HIV/AIDS cohort included were: (1) age exceeding 18 years, (2) individuals with a confirmed HIV diagnosis according to the “Chinese guidelines for diagnosis and treatment of HIV/AIDS” (AIDS and Hepatitis C Professional Group Society of Infectious Diseases CMA and Chinese Center for Disease Control and Prevention, 2018), and (3) those who were not undergoing cART. After enrollment, patients underwent follow-up at three to six-month intervals, including routine clinical examinations and blood sample collection. Individuals with HIV/HBV coinfection, recruited from this HIV/AIDS cohort, were required to be HBsAg positive for no less than six months and initiate cART based on the guidelines’ recommendations as specified in the study’s protocol. Throughout the follow-up, antiretroviral intervention could be either halted, initiated, or modified per prevailing clinical norms, as determined by the attending physician. The enrolled individuals had undergone a minimum of one year of on-treatment follow-up. The primary endpoint of this study was HBsAg loss. Data collected from January 2019 to September 2023 were included in the analysis.

HBV Mono-infected Cohort: Participants were recruited from outpatients at the Hepatology Unit, at Nanfang Hospital, between January 2019 and September 2023. The inclusion criteria for this group were non-cirrhotic CHB patients who tested positive for HBsAg for a minimum of six months and had initiated NAs therapy (Entacavir or Tenofovir disoproxil fumarate)following international guidelines. Enrolled patients had regular evaluations of virological and serological parameters related to HBV, conducted at intervals of no more than 6 months. Data collected retrospectively from 2019 to 2023 were included in the analysis.

Individuals in both groups were excluded if they showed signs of autoimmune hepatitis, alcoholic liver disease, tested positive for hepatitis C or hepatitis D viruses, had concurrent alcohol misuse, malignancies, a history of liver transplantation, other liver diseases, or cancer. All participants provided informed consent.

### 2.2 Clinical and laboratory evaluation

The quantification of HBV DNA was carried out utilizing a domestic HBV DNA assay kit (Daan Gene Co, Ltd.; Sun Yat-sen University; Guangzhou, China) in strict accordance with the manufacturer’s instructions, with a lower limit of detection of 1000 copies/ml. Serological markers were quantitatively determined using an ARCHITECT I2000SR (Abbott Ireland Diagnostics Division). The HBsAg test featured a lower limit of detection of 0.05 IU/ml. HBsAg loss was defined as a serum HBsAg concentration of less than 0.05 IU/ml. Serum alanine aminotransferase (ALT) and aspartate aminotransferase (AST) levels were determined at local laboratories, following standardized procedures. In this study, we calculated the AST-to-platelet ratio index (APRI) and the fibrosis index based on four factors (FIB-4).

HIV-1 RNA quantification was performed using the Roche RT-PCR assay, following the manufacturer’s instructions (Easy Q; Roche; lower limit of detection, 40 IU/mL). The Cells were stained with a panel of cell markers (FITC-CD4, CD8-PE, and CD3-PercP) and CountBright absolute counting beads. The cells were acquired on a BD FACS Canto II flow cytometer and the CD4+ and CD8+ lymphocyte counts were analyzed with FlowJo software. Antibodies and CountBright absolute counting beads were ordered from BD Biosciences.

### 2.3 Enzyme-linked immunospot

Peripheral blood mononuclear cells (PBMCs) were isolated by the Ficoll-Hypaque centrifugation and cryopreserved. PBMCs were resuscitated and rested in a 37°C incubator overnight. PBMCs stimulated with Core peptide pools in RPMI medium 1640 complete medium (Gibco; Thermo Fisher Scientific) supplemented with 10% FBS (Fetal Bovine Serum). After PBMCs were added with 18-mer overlapping peptide pool covered C open reading frame (2 μg/mL, GL Biochem, Shanghai, China), CD49d (1 μg/mL; Biolengd), and CD28(1 μg/mL; Biolengd). Cells were then cultured for 10 days with recombinant IL-2 (10 ng/mL; PeproTech) added on days 3 and 7. Cells were collected, washed 3 times on day 10, and rested in a 37°C incubator for 24 hours. Following stimulation again with the Core peptide, IFN-gamma cytokines secretion of HBV-specific T cells was detected according to the manufacturer’s instructions (Human IFN-γ ELISpot PRO; MabTech). T-cell responses to DMSO were included as a negative control in each assay. Spots on the plates were quantified using ImmunoSpot (Cellular Technology Ltd., Shaker Heights, OH). Results were deemed positive if the number of spots per well exceeded twice the negative control’s background level, with a minimum of 5 spots per well.

### 2.4 Statistical analyses

The follow-up duration was determined by calculating the time from the initial inclusion date to either the most recent recorded follow-up or the point at which the participant was lost to follow-up. Data were presented as either mean ± standard deviation (SD) or median (interquartile range). We compared group attributes using the χ2 test for categorical variables and employed the Student t-test or Mann–Whitney test for continuous variables. To minimize potential confusion in clinical characteristics between the two groups, we utilized the PS-matching method. This involved caliper matching using the nearest available matching method in the Propensity Score Matching (PSM), considering variables such as age, sex, HBsAg, and ALT.

Follow-up data were analyzed through Kaplan–Meier analysis and the log-rank test. The relationship between variables and endpoints was investigated using Cox proportional hazards regression analysis. The statistical software used was SPSS for Windows (version 26.0; SPSS Inc., Chicago, IL, USA) for all data analysis. The analysis included the implementation of the “survival ROC” package in R to assess the performance of CD4 and HBsAg through time-dependent ROC curve estimation. Additionally, the variation in CD4 and CD8 T cell count was computed from the initial evaluation to the final follow-up using a linear mixed effects model with the “nlme” package in R. A significance level of p<0.05 was applied for all analyses.

## 3 Results

### 3.1 Study population

The HIV/HBV coinfection rate in our cohort was 14.6% (170/2152). From the total of 170 individuals suffering from chronic hepatitis B, we eliminated the subsequent cases for analysis: 2 patients who showed positive results for anti-HCV, 1 patient diagnosed with diffuse large B-cell lymphoma, 1 patient with hepatocellular carcinoma, 10 patients with less than six months of follow-up, and 18 patients with incomplete clinical details (**Supplementary Figure 1**). A comprehensive examination was conducted on a combined total of 138 individuals with both HIV and HBV infections, who were monitored for a median period of 2.0 years. 129 (93.5%) patients initiated tenofovir disoproxil fumarate (TDF)+lamivudine (3TC)-based HBV therapy cART schedules, 8 (5.6%) used 3TC, and one received tenofovir alafenamide fumarate/emtricitabine. The demographic and clinical characteristics of 138 patients are summarized in **Table 1**. In the HBV Mono-Infected Cohort, 555 adult patients with HBV were initially screened from outpatients. Among them, 480 eligible patients were subsequently enrolled in this study (**Supplementary Figure 1**). 326 (67.9%) patients received entecavir (ETV),128(26.7%) received tenofovir disoproxil fumarate (TDF), 25 (5.2%) received tenofovir alafenamide fumarate (TAF). The clinical characteristics of the HBV mono-infection are detailed in **Table 1**.

**Table 1:** Clinical characteristics of HIV/HBV coinfected and HBV mono-infected individuals before and after propensity score matching.

	Before propensity score matching			After propensity score matching		
	HIV/HBV coinfection	HBV mono-infection	*P*	HIV/HBV coinfection	HBV mono-infection	*P*
n	138	480		99	155	
Age, year	35.37 ± 13.13	36.81 ± 9.01	0.312	38.28 ± 11.41	37.87 ± 9.43	0.786
Male, n (%)	127 (92.03)	347 (72.29)	<0.001	89(89.99)	132(85.16)	0.273
HBeAg-positive, n (%)	48(34.78)	304(63.33)	<0.001	41(41.41)	81(52.26)	0.092
HBsAg level, log_10_ IU/mL	3.18 ± 1.30	3.59 ± 0.81	<0.001	3.42 ± 1.29	3.43 ± 0.80	0.961
HBV DNA level, log_10_ copies/mL	4.45 ± 2.30	6.41 ± 1.65	<0.001	4.91 ± 2.34	5.65 ± 1.67	0.158
ALT, U/L	34.22 ± 40.15	155.80 ± 276.03	<0.001	40.57 ± 44.05	45.46 ± 48.47	0.414
AST, U/L	29.59 ± 21.97	106.78 ± 183.92	<0.001	32.42 ± 19.17	36.17 ± 20.83	0.193
TBIL, μmol/L	12.27 ± 8.63	20.12 ± 42.31	0.014	12.39 ± 9.67	17.84 ± 5.77	0.241
APRI score	0.44 ± 0.44	1.91 ± 4.34	<0.001	0.48 ± 0.46	0.58 ± 0.49	0.142
FIB-4 score	1.23 ± 1.34	2.52 ± 5.89	<0.001	1.23 ± 1.07	1.17 ± 0.75	0.636

ALT, alanine aminotransferase; AST, aspartate aminotransferase; APRI, the AST to-platelet ratio index; FIB-4, the fibrosis index based on four factors.

### 3.2 The rate of HBsAg loss in people living with HBV/HIV coinfection

**Figure 1:**
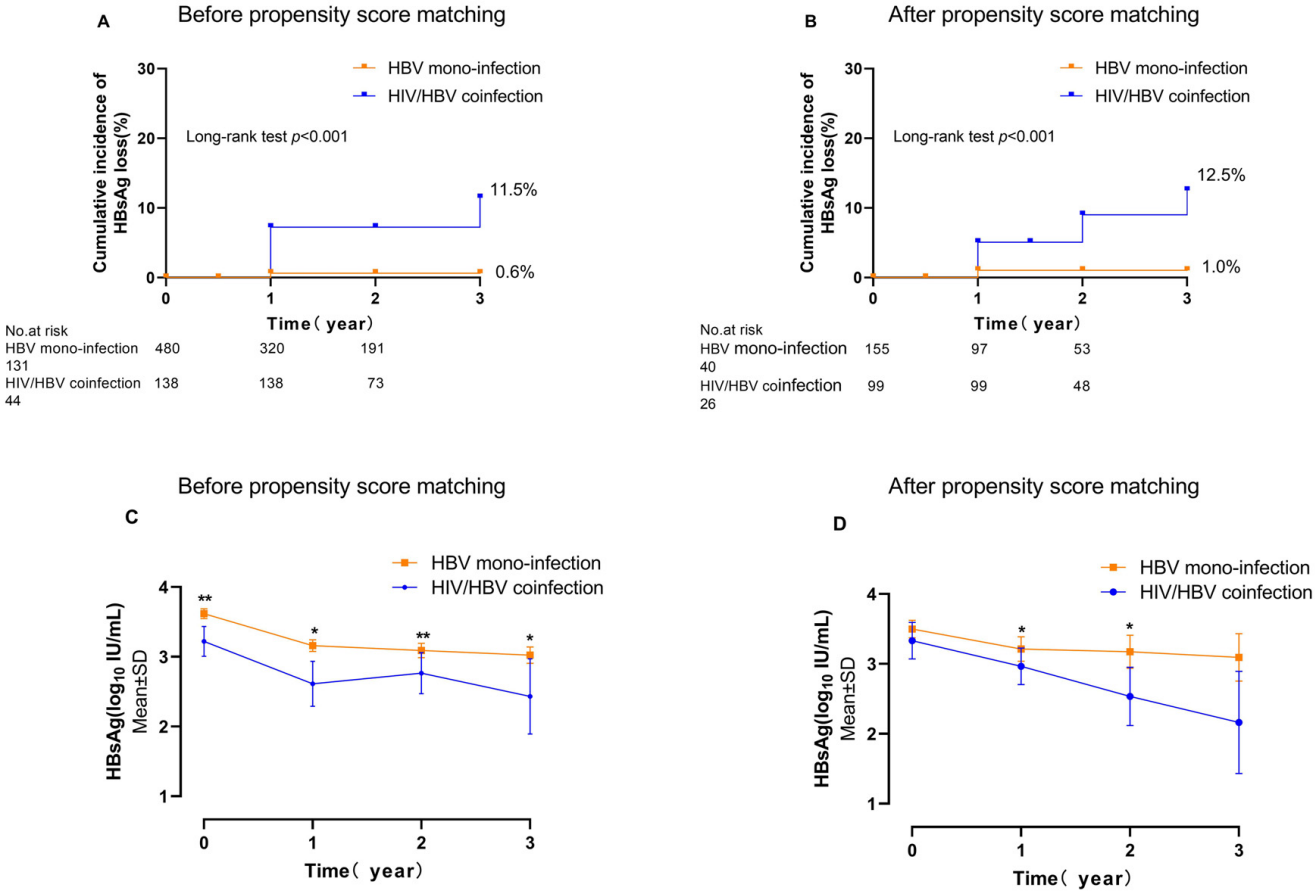
The cumulative incidence of HBsAg loss and the HBsAg kinetics in individuals with HIV/HBV coinfection compared to those with HBV mono-infection. **(A, C)** are overall individuals; **(B, D)** are propensity score-matched individuals. (*p<0.05, **p<0.01).

During enrolment, individuals in HIV/HBV coinfection were found to be younger compared to HBV mono-infection (35.37 vs. 38.28 years of age, *p*<0.001). A total of 13 patients achieved HBsAg loss with a 3-year cumulative incidence of 11.50% in HIV/HBV coinfection. In contrast, only 1 patient in the HBV mono-infection group experienced HBsAg loss, leading to an incidence of 0.6% (*p*<0.001) (**Figure 1A**). At year 1, the levels of HBsAg were notably reduced in individuals with HIV/HBV coinfection compared to those with HBV mono-infection (2.61 vs. 3.16 log_10_ IU/ml, *p*=0.001). This trend continued at year 2 (2.77 vs. 3.09 log_10_ IU/ml, *p*=0.040) and year 3 (2.43 vs. 3.02 log10 IU/ml, *p*=0.035) (**Figure 1C**).

After applying the propensity score (PS)-matching technique, there were 99 individuals in the HIV-HBV coinfected category and 155 individuals in the HBV mono-infected category. At enrolment, no notable distinctions in clinical characteristics or levels of HBsAg were observed between the two groups (**Table 1**). The 3-year cumulative occurrence of HBsAg loss was considerably greater in individuals with HIV/HBV coinfection compared to those with HBV mono-infection (12.5% vs. 1.0%, *p*<0.001) (**Figure 1B**). The PS-matched groups were compared over 3 years, revealing a continuous decrease in HIV/HBV coinfection. The HBsAg level in the coinfection group was significantly lower than that in the HBV-mono-infection group at year 1 (2.96 vs. 3.21 log_10_ IU/ml, *p*=0.021) and year 2 (2.53 vs. 3.17 log_10_ IU/ml, *p*=0.040) (**Figure 1D**).

### 3.3 Predictors for HBsAg loss in the HBV/HIV-1 coinfected groups

**Figure 2:**
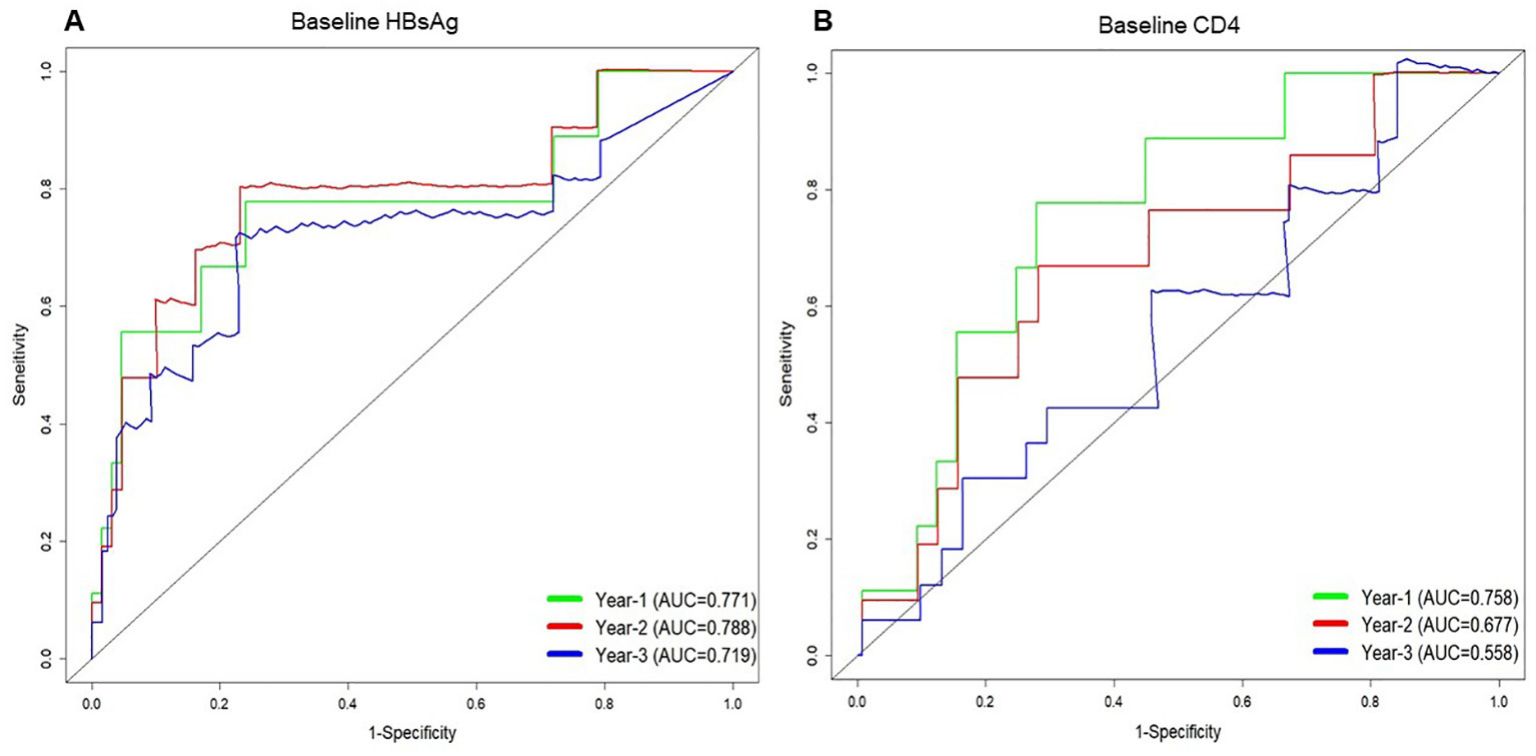
Time-dependent receiver operating characteristic (ROC) curves for predicting HBsAg loss at 1 year, 2 years, and 3 years based on the serum HBsAg level **(A)** and CD4 count **(B)** at baseline in people living with HIV/HBV coinfection.

**Figure 3:**
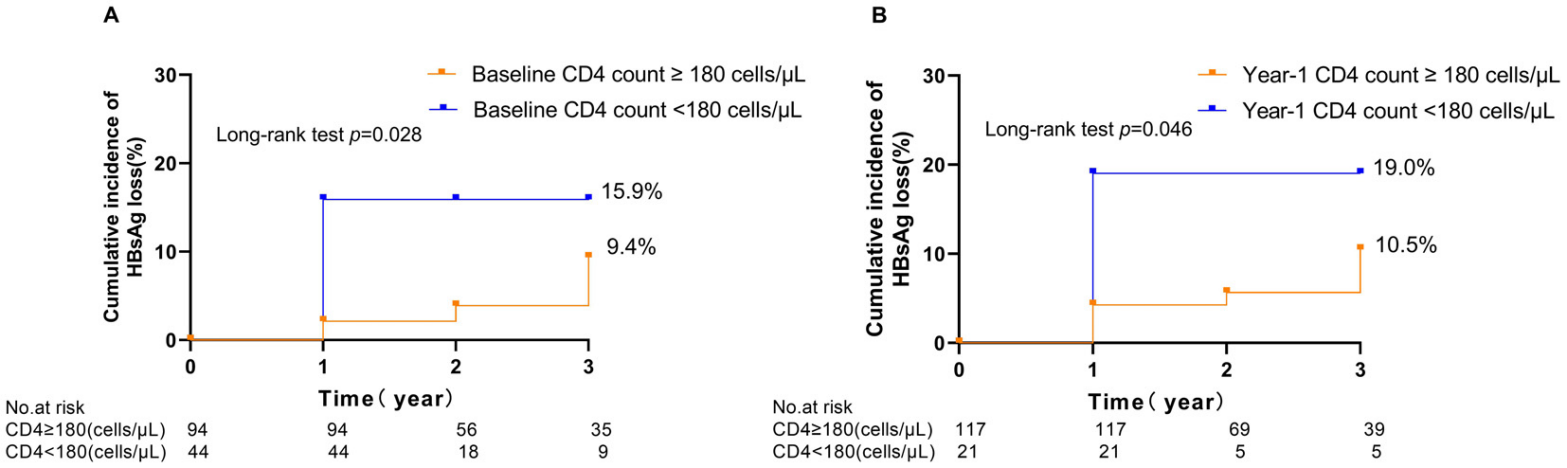
Kaplan–Meier curves illustrating the cumulative incidence of HBsAg loss following treatment in people living with HIV/HBV coinfection, stratified by CD4 cell counts at baseline **(A)** and 1 year **(B)**.

To further assess the predictive capability of variables at enrolment for HBsAg loss in the HIV/HBV coinfected groups. Multivariable analysis showed that lower HBsAg level (HR 0.53; 95% CI 0.38-0.74. *p*<0.001) and CD4 cell counts <180 cells/uL (HR 0.32; 95% CI 0.10-0.96. *p*=0.042) at baseline was associated with an increased indicator of HBsAg loss (**Table 2**). Furthermore, receiver-operating characteristic curve (ROC) curve analysis showed an area under the curve of 0.771 at year 1 for baseline HBsAg level and 0.758 at year 1 for baseline CD4 cell counts in predicting the incidence of HBsAg loss (**Figure 2**). In predicting HBsAg loss at year 1, the determined threshold for CD4 cell counts was set at 180 cells/uL (with a sensitivity of 78% and specificity of 72%). The baseline of CD4 cell counts <180 cells/uL were associated with the highest indicator of HBsAg loss as compared with the level ≥180 cells/uL (15.9%&9.4% at year 3, *p*=0.028, **Figure 3**). At year 1, CD4 cell counts <180 cells/uL were associated with the highest indicator of HBsAg loss as compared with the level ≥180 cells/uL (19.0%&10.5% at year 3, *p*=0.045, **Figure 3**).

**Table 2:** Cox proportional hazards regression analysis of HBsAg loss in HIV/HBV coinfection individuals (n=138).

	HBsAg loss(n=13)					
	Univariable			Multivariable		
	HR	95%CI	*P*	HR	95%CI	*P*
Male sex	0.04	0.00-220.23	0.471			
Age, per year	0.99	0.93-1.05	0.702			
HBeAg positivity	0.92	0.30-2.80	0.877			
HBsAg levels, per log_10_ IU/mL	0.60	0.44-0.81	<0.001	0.53	0.38-0.74	<0.001
HBV DNA, per log_10_ copies/mL	0.92	0.69-1.23	0.568			
HIV RNA level, per log_10_ IU/mL	0.99	0.93-1.06	0.841			
ALT, xULN	0.61	0.16-2.43	0.486			
AST, xULN	0.38	0.05-3.05	0.365			
CD4 count, cells/ul	1.00	0.99-1.00	0.094			
CD4<180 cells/ul	0.35	0.12-1.05	0.061	0.32	0.10-0.96	0.042
CD8 count, cells/ul	1.00	1.00-1.00	0.804			
CD4/CD8 ratio	0.07	0.00-2.62	0.150			
APRI score	0.10	0.00-4.76	0.246			
FIB-4 score	0.45	0.12-1.67	0.232			

ALT, alanine aminotransferase; AST, aspartate aminotransferase; APRI, the AST to-platelet ratio index; FIB-4, the fibrosis index based on four factors.

### 3.4 Kinetics of CD4 and CD8 T cell count

Following treatment, our study observed an increase in CD4 cell counts and a decrease in CD8 T cell count in people living with HIV/HBV coinfection. Notably, it was found that CD4 cell counts significantly increased (249.4 to 389.1, p<0.001) and CD8 T cell count significantly declined (978.1 to 733.4, p<0.001) from baseline to 1 year. Individuals with HIV/HBV coinfection experienced an annual rise of 65.9 cells/μL in CD4 cell counts. In comparison, CD8 T cell count in the same cohort declined by 110.4 cells/μL per year after cART (**Supplementary Figure 2**). There was no significant difference observed in the CD4 cell counts and CD8 T cell count between patients with HBsAg loss and those without HBsAg loss groups after cART (**Supplementary Figure 2**).

### 3.5 HBV-specific T response

**Figure 4:**
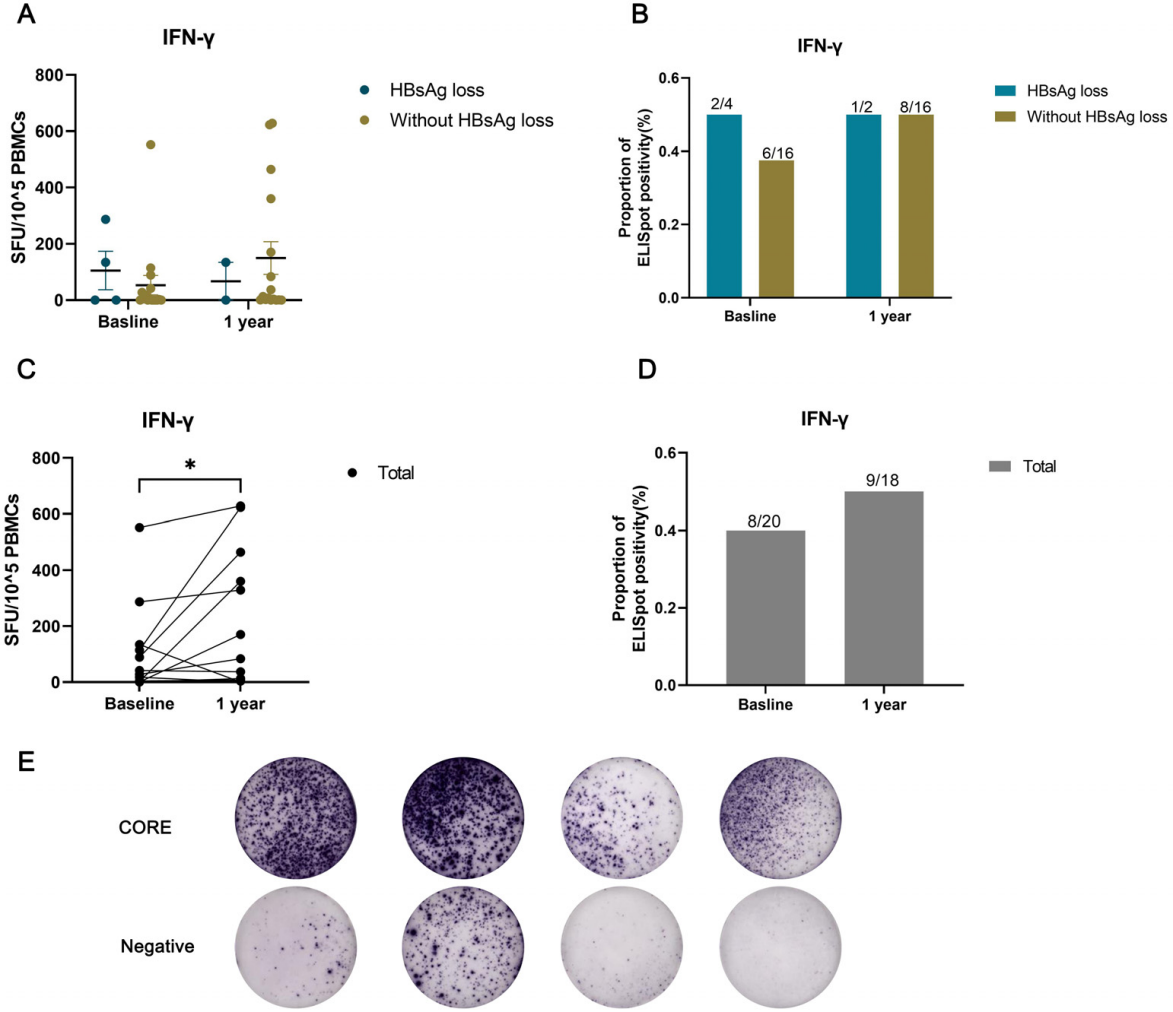
HBV-specific T-cell responses assessed using an IFN-γ ELISpot assay in individuals with HIV/HBV coinfection. The number of spot-forming units (SFU) per 10^5 PBMCs was compared between the HBsAg loss and without HBsAg loss groups **(A)**, or **(C)** across all patients at baseline and after one year of treatment. The proportion of ELISpot positivity was compared between the HBsAg loss and without HBsAg loss groups **(B)**, or **(D)** across all patients at baseline and after one year of treatment. **(E)** Representative image showing the results of the ELISPOT assay. (*p < 0.05).

To further assess immunological factors in individuals with HIV/HBV coinfection, we measured IFN-γproduction by HBV-specific T cells(**Figure 4**). Due to the limited availability of PBMC samples, a total of 20 patients were tested at baseline, 4 of whom experienced HBsAg loss. After 1 year of treatment, 18 patients were tested, with 2 showing HBsAg loss. HBV-specific T-cell responses exhibited a possible increasing trend one year after cART initiation compared to baseline (**Figure 4C**, *p*<0.05; **Figure 4D**, *p*>0.05). However, there was no significant difference in T-cell responses between the groups with and without HBsAg clearance (**Figures 4A, B**).

## 4 Discussion

This prospective study highlights the significance of quantitative HBsAg and CD4 cell counts in people living with HIV/HBV coinfection. Specifically, baseline CD4 cell counts of less than 180 cells/uL and HBsAg levels were associated with an increased indicator of HBsAg loss, even after adjusting for other important co-factors. The defined threshold of 180 cells/uL for CD4 cell counts in predicting HBsAg loss at one year demonstrated a sensitivity of 78% and a specificity of 72%.

In HBV mono-infected patients, the loss of HBsAg induced by treatment strongly correlates with a decreased likelihood of developing hepatocellular carcinoma (Kim et al., 2014; Yip et al., 2019). Therefore, the prognosis of individuals coinfected with HIV/HBV may be improved by the loss of HBsAg. However, in HBV mono-infected patients, oral antiviral treatment seldom leads to HBsAg loss (Yeo et al., 2019; Zhou et al., 2019; Hsu et al., 2021; Hsu et al., 2022). In our study, compared to those with HBV mono-infection, HIV/HBV coinfected individuals exhibited a significantly higher rate of HBsAg loss. This observation is consistent with findings in other cohorts and systematic reviews, which reported HBsAg loss rates ranging between 3.0% and 22% in individuals with HIV/HBV coinfection (de-Vries-Sluijs et al., 2010; Jiang et al., 2019; Yeo et al., 2019; Audsley et al., 2020; Chihota et al., 2020). The use of cART in coinfected individuals may enhance the immune response against HBV, suggesting that controlling HIV replication with cART can lead to more favorable outcomes in cases of concurrent HBV infection.

Next, we conducted an analysis to examine the association of baseline clinical variables with HBsAg loss in people living with HIV/HBV coinfection. In the multivariable analysis, a lower baseline HBsAg level was found to be associated with a higher indicator of HBsAg loss, consistent with findings from previous studies and reviews (Yeo et al., 2019). This outcome is anticipated, as decreasing levels of HBsAg signal an eventual complete loss of HBsAg.

Furthermore, the multivariable analysis revealed that CD4 cell counts below 180 cells/uL were associated with an increased indicator of HBsAg loss. Research has shown that HIV infection results in the destruction of CD4+ T cells in the host’s immune system while impairing the quality of the hepatitis B virus (HBV)-specific T-cell response in the setting of HIV/HBV coinfection (Chang et al., 2009; Bekker et al., 2023). Our data indicates a significant increase in CD4 count from baseline to 1 year, and most instances of HBsAg loss also occur within the first year of cART. Additionally, HBV-specific T-cell responses exhibited an increasing trend one year after cART initiation compared to baseline. This suggests that the occurrence of HBsAg disappearance may align with immune reconstitution, leading us to hypothesize that this could be the underlying mechanism behind the improved HBsAg loss. Similar to our results, a study in Zambia showed that baseline CD4 cell counts <350 cells/mm^3^ were associated with increased chances of achieving a functional cure (Chihota et al., 2020). Xiaodi et al. also illustrated that lower CD4 cell counts and immune activation were correlated with a rapid HBsAg decline in people living with HIV/HBV coinfection following the initiation of cART (Li et al., 2023). Significant decreases in quantitative HBsAg (qHBsAg) and disappearance of HBsAg have been associated with increases in CD4 count following the initiation of antiretroviral therapy (ART), and the recovery of HBV-specific T-cell responses in the peripheral blood was observed with HBV-active ART (Lascar et al., 2003; Lascar et al., 2005; Zoutendijk et al., 2012). Fluctuations in liver enzymes during therapy for HBV mono-infection typically suggest an immune response against HBV and may serve as a potential indicator for HBsAg loss (Wong et al., 2018; Ghany et al., 2020). Recent studies have suggested that hepatic flare, induced by immune reconstitution-induced inflammatory syndrome, also holds similar prognostic significance in coinfected individuals (Yoshikawa et al., 2021; Iannetta et al., 2022). The abrupt restoration of adaptive immunity gives rise to immune reconstitution inflammatory syndrome, subsequently expediting the generation of safeguarding antibodies. However, we did not observe significant differences in ALT fluctuations in patients with HBsAg loss compared to those without. The reason for the higher initial rate of HBsAg loss in people living with HIV/HBV coinfection remains unclear, but it may be linked to immune reconstitution following the initiation of ART. Further immunological research is needed to elucidate the specific mechanism.

This study has several limitations. First, the occurrence of HBsAg loss was rare, which limited the number of covariates that could be included in the prediction models. Spontaneous clearance of HBsAg in adults typically occurs within 3-6 months following initial infection; however, this process may be delayed or prolonged in individuals with HIV-related immunosuppression. By including only participants who had been HBsAg-positive for at least 6 months, we may have unintentionally excluded individuals who might have spontaneously cleared the virus earlier, particularly those with HIV-related immunosuppression. While this inclusion criterion is consistent with current guidelines for diagnosing chronic HBV infection, it may not fully exclude the possibility of spontaneous HBV clearance, especially in those with low CD4 counts. Future studies with longer observation periods and more detailed data on the duration of both HIV and HBV infection are necessary to better distinguish between spontaneous and treatment-induced HBV clearance. Second, given that the study visits spanned six months to a year, the median time to HBsAg loss may have been overestimated. Additionally, the small number of ELISpot-positive patients restricts the robustness of the correlation and statistical analyses. More patients and advanced immunological assessments are needed to better elucidate the role of HIV-associated immune recovery in the disappearance of HBsAg. Furthermore, additional underlying mechanisms may be at play, which were not captured in this study.

In conclusion, our study sheds light on the dynamics of HBsAg loss in HIV/HBV coinfected individuals after cART. The significantly higher rate of HBsAg loss in this population compared to HBV mono-infected individuals underscores the potential impact of effective HIV control on HBV clearance. Baseline HBsAg levels and CD4 cell counts emerge as promising predictive factors, equipping clinicians with valuable tools for indicator assessment and treatment decision-making.

## Data Availability

The original contributions presented in the study are included in the article/**Supplementary Material**. Further inquiries can be directed to the corresponding authors.
