# Supplementary material for: Baseline HBsAg quantitative and CD4 T cell counts are associated with HBsAg loss in people living with HIV/HBV coinfection after combined antiretroviral therapy
Source: Front Cell Infect Microbiol. 2025 Feb 19;15:1381826. doi: 10.3389/fcimb.2025.1381826 (PMC11880231; doi:10.3389/fcimb.2025.1381826)

**Supplement Fig.1** The flow diagram of patients through the screening process.


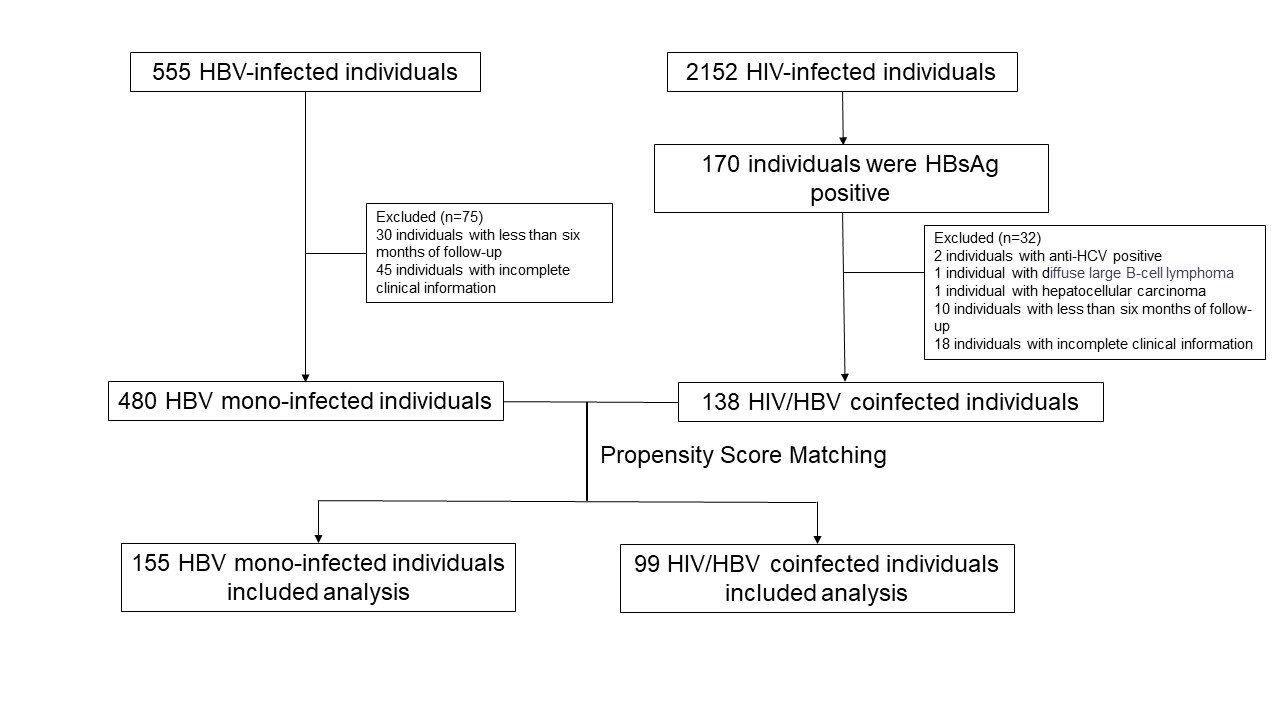


**Supplementary Fig.2** Using a linear mixed effects model, the dynamic changes in CD4 and CD8 T cell counts (A and C) in all patients with HIV/HBV coinfection after combined antiretroviral therapy (cART) further distinguish alterations in CD4 and CD8 T cell counts (B and D) in patients with HIV-HBV coinfection with and without HBsAg loss following cART. *** *p* < 0.001, ** *p*<0.01.
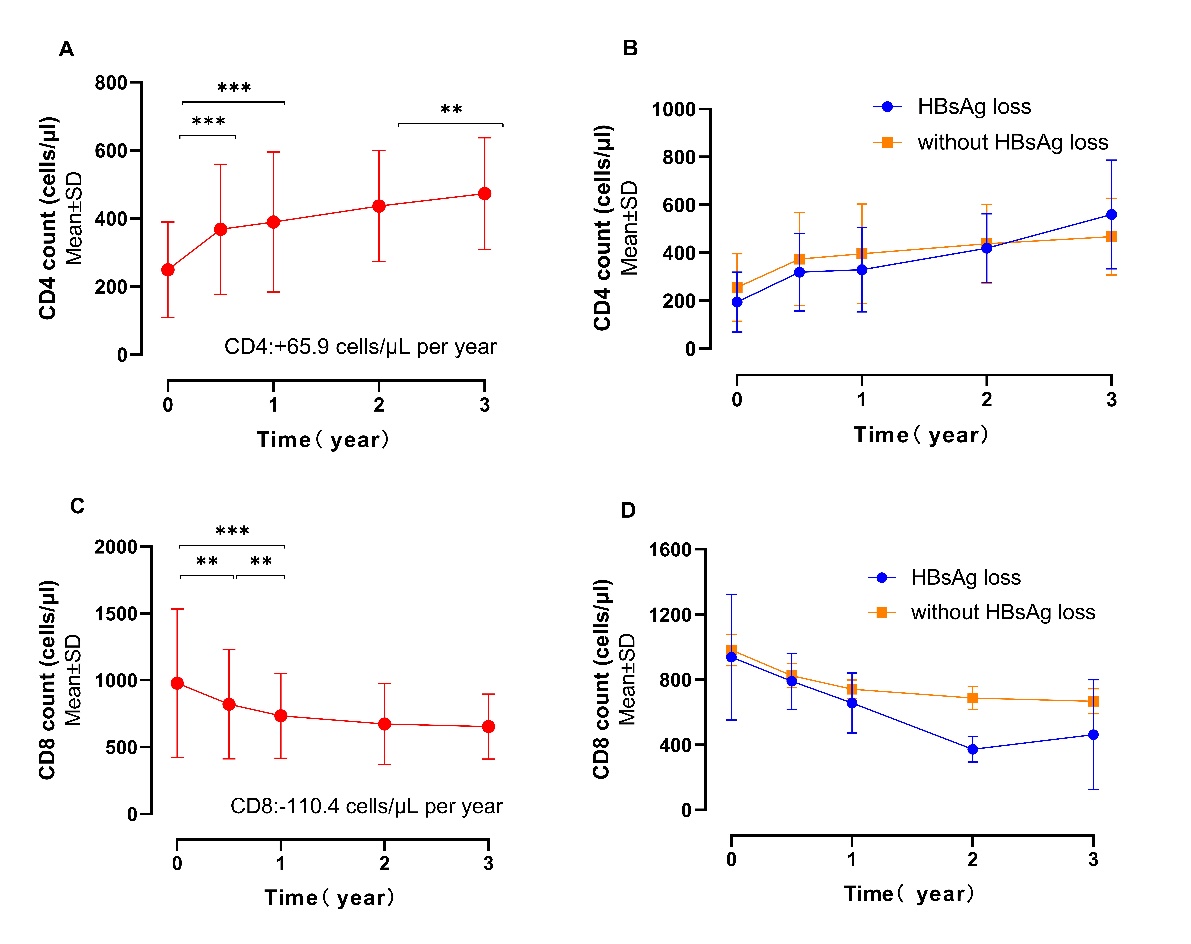

Supplement: Supplementary file 1 [file DataSheet1.docx]
